# Assessing CRISPR/Cas9 potential in SDG3 attainment: malaria elimination—regulatory and community engagement landscape

**DOI:** 10.1186/s12936-024-04996-x

**Published:** 2024-06-19

**Authors:** Aleksandra Snuzik

**Affiliations:** Jagiellonian University, Gołębia 24, 31-007 Kraków, Poland

**Keywords:** Malaria, Gene drive, CRISPR/Cas9, Target malaria, SDG3

## Abstract

Elimination of malaria has become a United Nations member states target: Target 3.3 of the sustainable development goal no. 3 (SDG3). Despite the measures taken, the attainment of this goal is jeopardized by an alarming trend of increasing malaria case incidence. Globally, there were an estimated 241 million malaria cases in 2020 in 85 malaria-endemic countries, increasing from 227 million in 2019. Malaria case incidence was 59, which means effectively no changes in the numbers occurred, compared with the baseline 2015. Jennifer Doudna—co-inventor of CRISPR/Cas9 technology—claims that CRISPR holds the potential to lessen or even eradicate problems lying in the centre of SDGs. On the same note, CRISPR/Cas9-mediated mosquito-targeting gene drives (MGD) are perceived as a potential means to turn this trend back and put momentum into the malaria elimination effort. This paper assessed two of the critical elements of the World Health Organization Genetically modified mosquitoes (WHO GMM) Critical Pathway framework: the community and stakeholders’ engagement (inability to employ widely used frameworks, segmentation of the public, ‘bystander’ status, and guidelines operationalization) and the regulatory landscape (*lex generali*, ‘goldilocks dilemma’, and mode of regulation) concerning mosquito-oriented gene drives (MGD) advances. Based on the assessment findings, the author believes that CRISPR/Cas-9-mediated MGD will not contribute to the attainment of SDG3 (Target 3.3), despite the undisputable technology’s potential. This research pertains to the state of knowledge, legal frameworks, and legislature, as of November 2022.

## Background

Malaria has been wreaking havoc on humanity ever since antiquity and remains so today [1]. This disease, with high morbidity and mortality in tropical and subtropical countries, is caused by parasites of the genus *Plasmodium* (and transmitted by a bite of an infected female mosquito of the *Anopheles* species [2–4]. Malaria elimination was a target set in General Assembly Resolution 55/2—widely known as the United Nations Millennium Declaration (goal 6, target 6C: “Have halted by 2015 and begun to reverse the incidence of malaria and other major diseases.”) [5] Even though it is widely accepted that the target 6C was achieved, malaria elimination and eradication [6] yet again [7] became a target for United Nations Member States in 2015, when resolution 70/1 Transforming Our World: the 2030 Agenda for Sustainable Development—widely known as the 2030 Agenda—was undertaken [8]. According to the Resolution’s Target 3.3, by 2030 member states aim to “end the epidemics of AIDS, tuberculosis, malaria, and neglected tropical diseases and combat hepatitis, water-borne diseases and other communicable diseases” [8].

Since the 2030 Agenda serves as a programming document, additional operational and technical papers were prepared to guide the efforts to attain the elimination and eradication objectives: Global technical strategy for malaria 2016–2030 (GTS) [9] and Action and Investment to defeat Malaria 2016–2030 (AIM) [10]. GTS sets four measurable goals accompanied by percentage-based targets: (1) to reduce malaria mortality rates globally by at least 90% compared with 2015; (2) to reduce malaria case incidence globally by at least 90% compared with 2015; (3) to eliminate malaria from at least 35 countries in which malaria was transmitted in 2015; (4) to prevent re-establishment of malaria in all malaria-free countries [9]. Meanwhile, AIM positions malaria in the border development agenda. It makes a case for the mobilization of resources by quantifying ROI and consolidating the evidence on the cost-effectiveness of malaria interventions [10].

The GTS-proposed endeavours are divided into three pillars and two supporting elements: Pillar 1) “Ensure universal access to malaria prevention, diagnostics, and treatment”; Pillar 2) “Accelerate efforts towards elimination and attainment of malaria-free status”; and Pillar 3) “Transform malaria surveillance into a core intervention”; Supporting element 1) “Harnessing innovation and expanding research”; Supporting element 2) “Strengthening the enabling environment” [9]. Under the umbrella of these pillars fell: implementing integrated vector management; using chemoprevention and chemoprophylaxis; ensuring universal diagnostic testing of all suspected malaria cases; providing quality-assured treatment to all patients; scaling up community-based diagnostic testing and treatment; enhancing pharmacovigilance and surveillance of the efficacy of anti-malarial medicines; protecting the efficacy of artemisinin-based combination therapy (ACT); eliminating *Plasmodium falciparum* malaria from the Greater Mekong subregion; removing all inappropriate anti-malarial medicines from markets (particularly oral artemisinin-based monotherapies); devising *Plasmodium vivax*-specific strategies; enacting new legislation. As per the supporting elements, GTS recounted new mosquito life-cycle targets (e.g., sugar feeding, mating, and oviposition phases); genetically modified mosquitoes, new species-specific point-of-care rapid diagnostic tests (RDTs) establishing the glucose-6-phosphate dehydrogenase (G6PD) status of patients; monitoring of molecular markers behind drug resistance development; and developing anti-malarial vaccinations, such as RTS,S/AS01 [9] (also known as *Mosquirix*), which received a positive scientific opinion from the European Medicines Agency in 2015 [11]. On the same note, AIM supplements the GTS's strategies by presenting good practices, case studies, and failures in the strategies' implementations. For instance, it depicts the already-taken endeavours for strengthening surveillance systems—Uganda's mTrac [12] and Zanzibar's Coconut Surveillance [13] programmes. Moreover, AIM addresses the determinants of malaria and pinpoints its potential sectoral matches, which could contribute to malaria elimination via collaboration [10].

Between 2015 and 2020, two additional initiatives were started as a response to levelling off the progress in malaria response. In 2016, the World Health Organization (WHO) identified 21 countries, spread across five WHO regions, most likely to defeat malaria (achieve zero indigenous malaria cases) within the 2020 timeline and launched the Eliminating Countries for 2020 Initiative (E-2020) [14, 15]. E-2020 undertakings were moderately effective [16].The second country-led initiative—High Burden to High Impact (HBHI)—was launched in 2018 by the WHO and Roll Back Malaria (RBM) Partnership to End Malaria [17] to support the 11 highest burden countries to “get back on track to achieve GTS 2025 milestones” [1]. These eleven countries—Burkina Faso, Cameroon, the Democratic Republic of the Congo, Ghana, India, Mali, Mozambique, the Niger, Nigeria, Uganda, and the United Republic of Tanzania—account for 70% of estimated malaria case burden and 71% of global estimated malaria-related deaths [1]. In 2020, out of these 11 countries, only India reported progress in reducing its malaria cases in 2020 [1].

## Need for change

Although implemented, 2015’s GTS strategies [9], E-2020 [14], and HBHI [17] initiatives did not bring us closer to attaining SDG’s target: malaria elimination. Paradoxically, despite the measures taken, the world faces a higher number of malaria cases than in the baseline 2015. Globally, there were an estimated 241 million malaria cases in 2020 in 85 malaria-endemic countries, increasing from 227 million in 2019 and compared with 2015 baseline of 224 million estimated malaria cases [1]. Malaria case incidence was 59, which means effectively no changes in the numbers occurred. Notably, the Covid-19 pandemic has taken its toll on the numbers due to the health service disruptions [20]. However, as showcased by the WHO, these disruptions were moderate: 78% of insecticide-treated bed nets (ITNs) from planned campaigns were distributed, seasonal malaria chemoprevention (SMC) was dispensed as planned, and indoor residual spraying (IRS) campaigns were also on target in most countries in 2020 [1]. Despite these endevours, the progress of reducing malaria morbidity and mortality objectively slowed, stalled, or even reversed in moderate- and high-transmission countries. This conclusion, coupled with increasing population, decreasing funding, waning political commitment, and most importantly, two major biological threats—deletions in the parasite’s *pfhrp2/3* genes, which render parasites undetectable by RDTs that are based on histidine-rich protein 2 (HRP2), and resistance to antimalarial drugs, as covered in the *Malaria Threats Map* [18]—force us to question the adopted strategy. Indeed, even the GTS strategies were reevaluated considering progress stalling [19].

Since the existing strategies need revision, and the calls for innovation are being voiced ever-lauder by various entities [20–24], it is wise to turn towards them. “*MalERA: an updated research agenda for malaria elimination and eradication”* pinpoints CRISPR/Cas9-mediated gene drives as new technology that will advance malaria biology [25]. On a similar note, Jenifer Doudna, the co-inventor of the technology, claims CRISPR holds the potential to lessen or even eradicate problems lying in the centre of SDGs [26]. Hence, this paper will evaluate CRISPR/Cas9-mediated gene drives’ potential to facilitate SDG attainment. To do so, two major elements of the WHO GMM Critical Pathway framework (hereinafter WHO GMM Guide) [27] are assessed: community and stakeholder engagement and regulatory landscape—concerning mosquito-oriented CRISPR/Cas9-mediated gene drives (hereinafter referred as “mosquito gene-drives” or MGD).

## Mosquito gene drives

The use of gene drives (GDs) in *Anopheles* went far past the proof-of-concept stage in 2015 when Gantz et al. established that genetic modification via CRISPR/Cas9-mediated GD in *Anopheles stephensi* leads to dual anti-*P. falciparum* effector genes introgression into ~ 99.5% of the progeny, following outcrosses of transgenic lines to wild-type mosquitoes [28]. Equally significant were the results obtained in 2016 by the Crisanti group, who using suppression CRISPR/Cas9-mediated GD in *Anopheles gambiae,* observed “a strong gene drive at the molecular level, with transmition rates to progeny of 91.4 to 99.6%.” [29]. Two years later, Crisanti's laboratory demonstrated that a CRISPR/Cas9-mediated GD targeting *doublesex* causes a complete population suppression in caged *An. gambiae* mosquitoes. Via blocking the formation of functional *AgdsxF*, they managed to reduce egg production to total population collapse in 7 to 11 generations [30].

While numerous research groups work on optimizing and standardizing MGDs-centered procedures [31], modelling studies report on MGDs anticipated efficiency and efficacy [32]. In addition to the modelling, the cost-effectiveness of MGD targeting *driving-Y* in the Democratic Republic of Congo (RDC) was assessed. Using a spatially explicit, agent-based model of malaria transmission in eight provinces in the RDC, Metchanun et al*.* determined that such intervention—providing that the GD is highly effective with at least 95% X-shredder efficiency—is the most cost-effective intervention (out of the combinations of ITNs and ACT) with the associate cost of deployment below 7.17 $int per person per year [33]. Aside from the abovementioned advances, entomological efficiency (entomological endpoints—particularly vectorial capacity and surrogates for entomological inoculation rate (EIR) [34]), monitoring requirements [35], testing-sites locations [36], and the target product profiles (TPPs) [37] were proposed.

Since it is possible to determine the preferred product characteristics (PPCs) [34] and TPPs [37] for MGDs, a shift to the phase-2 trials—understood as in the WHO GMM Guide [27]—stands open, at least from the Science perspective. Among multiple research groups concentrating on MGDs, *Target Malaria: a vector control research alliance* (hereinafter referred as “Target Malaria” or TM) holds a prominent position, operating in four African countries—Burkina Faso, Ghana, Mali, and Uganda—since 2005 [37]. Due to high-profile, large-scale, and well-documented pioneering research, TM is considered a trailblazer in MGD research. For the same reasons, TM undertakings are the point of reference throughout this paper.

## Target malaria

TM has been following a phased pathway with three stages of research [38], based on the guidelines from the WHO GMM Guide [27], the 2016 National Academies of Sciences, Engineering and Medicine (NASEM) Report "Gene Drives on the Horizon" [39] and the 2018 “Pathway to deployment of gene drive mosquitoes as a potential biocontrol tool for elimination of malaria in sub-Saharan Africa: recommendations of a scientific working group” [40]. The first stage of TM development pathway, “*Sterile man*”, started in 2008 and was concluded in 2021. This stage involved non-GD genetically modified (GM) sterile male *An. gambiae* research, in containment, in Burkina Faso and Mali and small-scale release of hemizygous, non-GD GM *Anopheles coluzzii* in Bana Village, Burkina Faso [41]. Based on the findings from the mark-release-recapture experiment conducted within 20 days from the release, it was determined that the intervention reduced fitness and mosquito dispersal [42]. The second phase of the research, titled "Male bias", focuses on fertile non-GD *An. gambiae*, genetically modified to produce mainly male mosquitoes (up to 95% in the laboratory) [43]. In March 2022, modified and intercrossed strains were shipped from Italy to Burkina Faso [44]. No stage-2 release date has been announced yet. The purpose of the first two steps is to better inform the development pathway of step 3: "*Male bias and female fertility*", entailing the release of self-sustaining MGDs. The focus of this stage is still being investigated. However, TM stated two of the most promising options: (1) “A genetically modified strain with fertile males that produce predominantly male offspring, leading to a distortion in the sex ratio of the targeted mosquito population”; (2) “A genetically modified strain with fertile males carrying a gene that will spread through the mosquito population and cause females that inherit the gene from both parents to be sterile” [45]. No start date for stage 3 research has been announced so far. However, it is said that the project intends to test MGD in sub-Saharan Africa as early as 2024 [46].

## Regulatory landscape

A factor that could hinder CRISPR/Cas9-mediated GD's potential for malaria elimination is the regulatory landscape. Even though field-based evaluations of genetically-modified organisms (GMOs) are not without precedent—e.g., *Wolbachia*-infected mosquitoes [47]—the MGD release does not have any exact equivalent [48]. To date, no legally binding provisions on the international level have been established regulating specifically MGDs.

In the absence of *lex specialis*, *lex generali—*in this case, the Convention on Biological Diversity (CBD) [49] and its protocols the Nagoya Protocol on Access and Benefit-sharing [50], the Cartagena Protocol on Biosafety [51], and the Nagoya- Kuala Lumpur Supplementary Protocol on Liability and Redress to the Cartagena Protocol on Biosafety [52])—serves as a primary source of legislation for its signatories—countries that transposed or implemented the provisions into national law. Based on the CBD Report of the Ad Hoc Technical Expert Group on Synthetic Biology in 2017, organisms containing GD fell under the definition of living-modified organisms (LMOs) as per the Cartagena Protocol on Biosafety [53, 54]. Even though a CBD ban on releasing organisms carrying GD was proposed twice—in 2016 and 2018 [46, 55]—it did not come into force. Based on the decision CBD-COP14—undertaken on the 29th of November 2018, during the United Nations Convention on Biological Diversity in Sharm El-Sheikh, Egypt, three additional premises to be met before a release of GD organism were enforced: (1) “Scientifically sound case-by-case risk assessments have been carried out”; (2) “Risk management measures are in place to avoid or minimize potential adverse effects, as appropriate”; and (3) Where appropriate, the "prior and informed consent," the "free, prior, and informed consent" or "approval and involvement" of potentially affected indigenous people and local communities is sought or obtained, where applicable in accordance with national circumstances and legislation” (CBD/COP/14/L.31) [56]. Other—relevant to the subject of gene drives—international entities such as the International Union for the Conservation of Nature (IUCN), WHO’s Vector Control Advisory Group (VCAG), the Malaria Policy Advisory Group (MPAG), and the Technical Advisory Group for Neglected Tropical Diseases, while issuing frameworks and guidelines, have no regulatory purview; hence, their regulatory endeavours come down to—at most—soft law.

In the WHO African Region, where the first MGD release is said to occur, genetically modified insects—such as MGD—“are regulated by Competent National Authorities whose mandate derives from national biosafety laws” [57]. The enactment of national biosafety laws is protracted and subject to political forces, which is best illustrated in the lack of biosafety law in Uganda [58, 59], where TM research takes place. African Region, under the leadership of the African Union (AU), has not enforced a coordinated regulatory framework for GD. Neither established harmonization of biosafety regulations in its 55 member states, even though such legislative endeavour was undertaken [60]. The African Union Development Agency, the New Partnership for Africa’s Development (AUDA-NEPAD), has been leading the way in the MGD regulatory process: organizing panels and regional consultations [61] and strengthening AU Member State’s regulatory capacities [62]. AUDA-NEPAD has also been mandated to oversee GD research, including field evaluations [57]. One of the AUDA-NEPAD programmes—the African Biosafety Network of Expertise (ABNE)—constitutes an opportunity to build a functional GD-regulatory system in Africa [63]. Notably, AUDA-NEPAD is a grantee of the Bill & Melinda Gates Foundation, which provides “core funding” to the Target Malaria consortium [64, 65]. Aside from NEPAD, two other organizations in Africa are involved with regulatory discussions of GD: the Pan-Africa Mosquito Control Association and the Africa Academy of Sciences [66]. Other countries—such as the USA via the Foundation for the National Institutes of Health (FNIH)—have supported African countries in their regulatory capacity. This support, however, is limited to running regulatory workshops [67].

As per the transboundary movement of GMOs, most malaria-endemic countries where MGD may be released are signatories to the Cartagena Protocol on Biosafety to the CBD. Importantly, all the counties where Target Malaria research is conducted—Mali, Burkina Faso, Uganda, and Ghana—are parties of CBD, and all its protocols, except for Ghana, which is not a party of the Nagoya—Kuala Lampur Supplementary Protocol on Liability and Redress [52]. Considering Target’s Malaria phased research design, the Cartagena Protocol—particularly Article 17 on transboundary movement of LMOs [51]—is of importance since the GMM movement already took place in 2016 when the national competent authority—National Biosafety Agency (ANB) of Burkina Faso—authorized Target Malaria to proceed with the 1st stage of TM research and import transgenic mosquito from Italy to Burkina Faso for the first phase of TM project [68]. In 2021, ANB of Burkina Faso again issued such authorization for the second phase of the TM project. The male bias mosquitoes were developed in the UK, bred, tested in Italy and the USA, and finally imported to Burkina Faso on the 16th and 21st of March 2022 [69].

The legislative landscape of one of the countries pioneering the GD technology—the USA is particularly complicated. It is such for two reasons: USA is neither a party of CBD nor any of its protocols, nor is the jurisdiction over MGD not clearly stated. In the USA, GMOs and subsequent MGD fall under the Coordinated Framework for the Regulation of Biotechnology, including three agencies—the Food and Drug Administration (FDA), the Department of Agriculture (USDA), and the Environmental Protection Agency (EPA)—all of which could be of competent jurisdiction [39, 66]. Since the jurisdictional regulation is product-based, the final MGD-product will determine the relevant regulatory agency. In the case of arthropods-oriented GD, the anticipatory lead regulatory oversight for the release of MGD, intended to reduce or interrupt disease transmission, might be considered through FDA in conjunction with the USDA [70]. However, MGD products that intend to function as a pesticide for population control fall into the jurisdiction of the EPA [71]. This regulatory flux has already caused controversy when the regulatory jurisdiction over Oxitec’s *Aedes aegypti* field trial was changed from the FDA to EPA [72, 73]. In 2020, the Department of Agriculture Animal and Plant Health Inspection Service released the final rule amending the regulations of the movement of genetically engineered organisms (including MGD) [74]. This amendment is significant considering the potential transboundary movement to/ from malaria-endemic countries of mosquitoes intended for open trials [66].

As recounted, no MGD-oriented *lex specialis* has been developed on the international level. Such an omission in the form of an *intra-lege* legislation gap creates a state of legislative flux, impeding the investments and protracting the research process. The protractedness of enacting MGD legislation stems from problems of different natures. One of the most prominent is the overlap of the organizational relevance to the regulation of MGD research. Notably, two of the most authoritative stakeholders of the GD governance—WHO and CBD—hold different premises for values and their orientations towards GD: WHO's focus on public health does not seem to go in hand with the CBD's focus on biodiversity conservation [48]. While their guidance does not have to be contradictory, the international legislative body must strike a balance and satisfy the dual priorities of minimizing the risk to the environment and demonstrating large-scale positive public health impact. To resolve this problem, Governance Coordinating Committees (GCCs) were proposed as an international governance body [75]. According to Kelsey et al*.*, the GCC framework should consist of four essential constituent bodies: (1) the source country, (2) the international organizations or governing bodies (WHO/UN Environment Programme UNEP), (3) the DEC (disease-endemic country) governing authorities, and (4) the DEC local organizations [66].

The second major problem the potential MGD legislative process faces is indecisiveness as per the mode of regulation of MGD (in juxtaposition other with regulations covering LMOs.) On a national level, the regulatory status of GD varies from “Lightly Regulated” through “Regulations in Development” and “Highly Regulated” to “Prohibited” [76], as noted in the Global Gene Editing Regulation Tracker—compiling GD-related legislation of 24 countries and regions. Literature enumerates the negatives of regulating MGD differently from other LMOs, primally due to the exceptionalization of MGD regulations leading to conditions in which novel technologies would consistently require unique governance strategies that would be difficult to meet [66]. These multiple regulatory approaches are not conducive to reaching a consensus on the international level, rendering legislative efforts—particularly the treaty negotiation phase—ineffective. Moreover, due to the transboundary nature of MGD, the legal effects of reservations and objections—understood as in the Vienna Convention on the Law of Treaties—is of little consequence [77]. The same applies to article 34 of the Convention on the legal effect of a treaty for any third State [77].

Another regulatory-oriented problem concerns the ‘Goldilocks dilemma’ regulators worldwide are facing [78]. If the regulators are too precautionary, they will err on the safe side, keeping the MGD from the burdened populations and thus making type I error [79]. Conversely, if they are insufficiently cautious, MGD could pose significant harm to already afflicted populations, making type II error. Striking a balance on the ‘Goldilocks fulcrum’, as described by Rudenko et al*.* [80], should be placed at a predictable balance between type I and type II errors. If regulators were to find the focal point based only on scientific evidence—as it is in the USA—the fulcrum placement would be *"a function of posing the appropriate risk question and answering them to the extent possible." *[80]. Aside from the fact that insufficient predictions for environmental risk assessment (ERA) were generated—particularly in the case of population suppression drives—it is impossible to navigate regulations solely on the scientific data. As noted by Rudenko et al., science-based concerns—regardless of under which regulatory rubric they occur—“intrinsically contain value judgments that may be cryptic to those unfamiliar with regulatory risk assessment” [80, 81]. Hence, MGD-concerning regulatory decisions will never be based solely on science. Bearing in mind the 'Asilomar in memory' fiasco, as described by Hurlbut [82], the regulatory process should have more levers. In the case of MGD, regulation—understood as a rule of order having the force of law prescribed by a superior or competent authority—is not enough. It is governance—the process of interaction throughout an organized society's laws, norms, power, or language—a *conditio sine qua non* in the MGD-oriented legislative process.

## Community and stakeholder engagement

Any form of governance needs input legitimacy. In the case of CRISPR/Cas9-mediated MGDs, the input legitimacy is delivered via community and stakeholder engagement (CSE). Learning from the ‘Asilomar fiasco’ [82], the relation to native and disenfranchised communities in the MGDs development process must be significant to ensure its success. However, due to CRISPR/Cas9-mediated GDs no prior use history and area-vide nature of the intervention entailing risks on a collective level, CSE endeavours pose a great challenge. Firstly, MGD nature renders widely recognized frameworks for CSE—the Declaration of Helsinki [83], the International Ethical Guidelines for Health-Related Research Involving Humans [84], or the Free, Prior, and Informed Consent [85] impractical to employ directly [86].

Secondly, a major theoretical problem still needs to be resolved: segmentation of the public and the 'bystander' status. As noted by Rudenko et al., “the terms 'public' and 'stakeholders' typically have been poorly defined and have often been lumped together anyone who was not involved in the actual production of a biotechnology product” [80]. Such a take on the definition bifurcates the population into 'scientists' and 'public'. Even MGD regulatory entities—such as WHO [27] and NASEM [39]—do not share a common understanding of the segmentation of the public [80]. In parallel, TM, the Kenya Medical Research Institute (KEMRI) and the Pan African Mosquito Control Association (PAMCA) developed their understanding of the terms 'relevant communities and stakeholders' [87]. They distinguished five areas of differing stakeholder engagement: (1) “village with mosquito release”; (2) “village with mosquito collection”; (3) “village with no entomological activity”; (4) “neighbourhood surrounding insectary for contained use”; and (5) “other stakeholders outside of the monitoring area” [87]. While such a delineation of relevant stakeholder communities presents a step forward to CSE development, it is controversial. It excludes four out of the five mentioned groups from consenting to the GMD release. In the short term, this issue is particularly relevant to the communities that are 'bystanders' as understood by Walen [88]. However, in the longer perspective, and due to the MGD persistence in nature and its predicted spatiotemporal spread, this issue will also apply to other groups. Based on the proceedings of an expert workshop on community agreement for gene drive research in Africa co-organized by KEMRI, PAMCA, and TM, the other 'public' groups, could be approached by researchers in "a more devolved or mediated models" for example through local authorities to broker community agreement [87]. Moreover, however sound, the theoretical delineation proposed during the workshop may only be partially translatable into practice. Since the spatiotemporal spread of MGD for field trials is unknown, changes in the predicted rate of geographical and temporal dispersal of MGD may have profound implications on the CSE segmentation. This could be the case, especially when the dispersal is great enough so that affected communities do not share mutual interests.

Another problem MGD-oriented CSE faces is the abstract nature of all relevant guidelines. As aptly noted by Hartley et al., "There is frequent slippage to a reductive rendering of engagement as the right thing to do or as a way to secure public acceptance." [89]. Such programming and general notions permeate from WHO GMM [27] and NASEM [39]. These guidelines deliver all but detailed instructions as per the scope of desirable CSE undertakings. The only issue—generally accepted as resolved—is the WHO stance presented in the GMM Guide settling the dispute of whether individual consent is needed for the open release of MGD mosquitoes [27]. Unless there is a collection of samples or data from human participants, the collection of individual consent is not needed. Instead, the WHO calls for 'community authorization' prior to MGD mosquitoes [27]. No consensus on interpreting the term 'community authorization' has been established. However, a major step forward in this realm can be attributed to TM, which operationalized these guidelines. The operationalization took place on many levels: adapting to stakeholders' preferences, inclusiveness, empowerment, and accountability. The interpretation of the term 'community authorization' could be inferred from that operationalization [90]. TM's undertakings include obtaining consent for routine entomology collections, conducting entomology research with the active participation of the local communities [92], conducting longitudinal ethnographic studies to inform the communities' governance landscape [91], organizing meetings with the public and the researchers in the form of 'café citoyen' ('citizen café'), involving a traditional local system of organizations in CSE activities, running focal groups and Test and Treat Day activities, organizing a 'relay group' to frame structured interactions with civil society representatives, co-establishing with the community monitory committee, devising community acceptance model, and running qualitative verification studies [90]. Early in the engagement strategy design, an internal engagement audit was conducted to ensure the CSE activities had been carried out in compliance with the project engagement strategy, to evaluate whether communities and stakeholders were "ready" for the activity, and finally, to consider the preparedness of the project team and its systems to manage the activity [91].

To date, some critical voices about TM's CSE undertakings have been voiced, particularly about the lack of fully informed consent [93, 94]. Vekcha claims that "Target Malaria obtained the signature of the Bana village community acceptance form from people who do not understand anything about the project and who do not realize the issues of their signature." [93]. A representative from Bana Village, Burkina Faso, where many of the CSE undertakings took place, noted, "*They* [TM Researchers] didn't tell us about the risks, only the advantages" [95]. Looking closely at these claims, one cannot accept them indiscriminately. In fact, they stand—at least partially—in contradiction with the results of the study conducted in Bana as per the comparative picture of community knowledge on mosquitoes and malaria in 2014 and 2019 [91]. Other allegations were put forward by African Centre for Biodiversity and Gene Watch, questioning the rationale behind community engagement in the research: financial incentives to participate in the research [96]. As showcased by Barry et al., the financial incentive was one of the five major motivations behind participation in TM's CSE [92].

## Conclusions

As showcased throughout this work, CRISPR/Cas9-mediated mosquito-gene drives hold significant promise for malaria elimination, thereby potentially aiding in attainment of the Target 3.3 of Sustainable Development Goal no 3. However, due to the controversial nature of this technology and its operating context, stand-alone scientific advances are insufficient to secure such formidable accomplishment as making the world a malaria-free zone. Two of the critical elements of the WHO GMM Critical Pathway framework—community and stakeholder engagement and regulatory landscape—could greatly hinder MGD's potential. Indeed, these factors will very likely impede the progress of MGD development. Even though, as available data indicate, the first open field releases of MGD will take place in this decade, worldwide adoption of this technology is not expected to occur due to protracted legislation process, political controversies, and potential fallbacks from unauthorized—at the international level—employments of this technology, just as it was with the case of GMO. With the Goldilocks dilemma at the heart of problems within the regulatory process, it is imperative to address the community and stakeholders' engagement strategies in the timeliest manner possible. Albeit significant work in the operationalization of CSE was done by Target Malaria, their CSE undertakings should be addressed and subjected to scientific scrutiny. Both CSE developed under the scrutiny of science and protracted-at-its-core international legislative process take time. The deadline for meeting the SDGs targets is not adjustable, however. This said, it is the author's opinion that CRISPR/Cas-9-mediated MGD will not contribute to the attainment of the SDG3, despite the undisputable technology's potential.

## Data Availability

Not applicable.

## Abbreviations

ABNE: African Biosafety Network of Expertise
ATC: Artemisinin-based combined therapy
AU: African Union
AUDA-NEPAD: African Union Development Agency—New Partnership for Africa’s Development
CBD: The United Nations Convention on Biological Diversity
CRISPR/Cas9: Clustered regularly interspaced short palindromic repeats/ CRISPR-associated protein
CSE: Community and Stakeholder Engagement
DEC: Disease-endemic country
EIR: Entomological inoculation rate
EPA: Environmental Protection Agency
ERA: Environmental risk assessment
FDA: Food and Drug Administration
G6PD: Glucose-6-phosphate dehydrogenase
GCCs: Governance Coordinating Committees
GD: Gene drive
GMO: Genetically modified organism
GMM: Genetically modified mosquitoes
HBHI: High Burden to High Impact Initiative
HRP2: Histidine-rich protein 2
IRS: Indoor residual spraying
ITN: Insecticide-treated net
IUCN: International Union for the Conservation of Nature
KEMRI: Kenya Medical Research Institute
LMO: Living modified organism
MPAG: Malaria Policy Advisory Group
MGD: Mosquito-oriented (targeting) gene drive
NASEM: National Academies of Sciences, Engineering and Medicine
PAMCA: Pan African Mosquito Control Association
PPC: Preferred product characteristic
RBM: Roll Back Malaria Initiative
RDC: Democratic Republic of Congo
RDT: Rapid diagnostic test
SDG: Sustainable Development Goal
SMC: Seasonal malaria chemoprevention
TM: Target Malaria A Vector Control Research Alliance
TPP: Target product profile
VCAG: Vector Control Advisory Group
WHO: World Health Organization
WHO GMM: WHO GMM Critical Pathway Guide
UNEP: WHO/ UN Environmental Programme
USDA: United States Department of Agriculture
